# A Single-Granule-Level Approach Reveals Ecological Heterogeneity in an Upflow Anaerobic Sludge Blanket Reactor

**DOI:** 10.1371/journal.pone.0167788

**Published:** 2016-12-09

**Authors:** Kyohei Kuroda, Masaru K. Nobu, Ran Mei, Takashi Narihiro, Benjamin T. W. Bocher, Takashi Yamaguchi, Wen-Tso Liu

**Affiliations:** 1 Department of Civil and Environmental Engineering, University of Illinois at Urbana-Champaign, North Mathews Ave, Urbana, Illinois, United States of America; 2 Department of Environmental systems Engineering, Nagaoka University of Technology, Kami-tomioka, Nagaoka, Niigata, Japan; 3 Bioproduction Research Institute, National Institute of Advanced Industrial Science and Technology (AIST), Central, Higashi, Tsukuba, Ibaraki, Japan; 4 Petrochemicals Technology, BP America, Naperville, Illinois, United States of America; Fudan University, CHINA

## Abstract

Upflow anaerobic sludge blanket (UASB) reactor has served as an effective process to treat industrial wastewater such as purified terephthalic acid (PTA) wastewater. For optimal UASB performance, balanced ecological interactions between syntrophs, methanogens, and fermenters are critical. However, much of the interactions remain unclear because UASB have been studied at a “macro”-level perspective of the reactor ecosystem. In reality, such reactors are composed of a suite of granules, each forming individual micro-ecosystems treating wastewater. Thus, typical approaches may be oversimplifying the complexity of the microbial ecology and granular development. To identify critical microbial interactions at both macro- and micro- level ecosystem ecology, we perform community and network analyses on 300 PTA–degrading granules from a lab-scale UASB reactor and two full-scale reactors. Based on MiSeq-based 16S rRNA gene sequencing of individual granules, different granule-types co-exist in both full-scale reactors regardless of granule size and reactor sampling depth, suggesting that distinct microbial interactions occur in different granules throughout the reactor. In addition, we identify novel networks of syntrophic metabolic interactions in different granules, perhaps caused by distinct thermodynamic conditions. Moreover, unseen methanogenic relationships (e.g. “*Candidatus* Aminicenantes” and *Methanosaeta*) are observed in UASB reactors. In total, we discover unexpected microbial interactions in granular micro-ecosystems supporting UASB ecology and treatment through a unique single-granule level approach.

## Introduction

Upflow anaerobic sludge blanket (UASB) reactors are widely implemented as an effective biotechnology for treating various wastewaters types (*e*.*g*., municipal, industrial, and food-processing) [1]. Achieving high treatment efficiency requires formation of granular sludge with a function-coordinated microbial community to ensure biomass retention and pollutant degradation. In addition to reactor operation [2, 3], balance between three primary niches (syntrophs, methanogens, and fermenters) is known to be necessary for mineralization of organic pollutants to CH_4_ and CO_2_ [2, 4, 5] based on previous studies implementing 16S rRNA-based tools (e.g., high-throughput DNA sequencing) [6–9]. While such studies provide a “macro”-level view of the reactor ecosystem, fluorescence *in situ* hybridization (FISH) on individual granules has shown intricate microbial composition and spatial distribution in each granule [5, 10]. Based on such studies, we suspect that UASB reactors ought to be viewed as a composite ecosystem (reactor) of discrete micro-ecosystems (granules) and that we are far from unraveling the complex ecology and community development of granular biomass. Moreover, there are likely overlooked microbial heterogeneities between and within individual granules that can potentially aid us in better understanding UASB reactors as a biological ecosystem for future operational improvement.

Networks of microbial metabolic interactions in granules are especially critical as treatment of various compounds frequently found in wastewaters depend on multiple organisms. Specifically, to accomplish degradation of many compounds (*e*.*g*., fatty acids and aromatic compounds), specialized organotrophic *Bacteria* (syntrophs) and CH_4_-producing *Archaea* (methanogens) form intimate interactions, a phenomenon known as syntrophy [11]. In this study, we investigate UASB reactors treating wastewater from purified terephthalic acid (PTA) synthesis as a model because such reactors contain compounds (*e*.*g*., terephthalic acid–TA; benzoic acid–BZ) whose degradation is known to highly depend on metabolic networks and syntrophy [12–16]. Moreover, few studies have investigated bioreactors treating PTA wastewater [16–18], despite its copious production due to the extreme global demand for purified terephthalic acid (PTA) as a building block for plastics and polyester fibers. We specifically analyze 300 individual granules from both lab- and full-scale PTA wastewater-treating UASB reactors using 16S rRNA gene sequencing to uncover the microbial interactions governing the micro- and macro-level ecology. In doing so, we discover unexpected diversity among granules and novel microbial networks in full-scale reactors and provide valuable insight into resolving the biological complexity of full-scale reactors, a major hurdle in improving wastewater treatment.

## Materials and Methods

### Characteristics of PTA wastewaters, reactor operations, and reactor performances

Reactors E and F are replicate reactors treating PTA wastewater with identical configuration, volume (4,562 m^3^), temperature (34°C), total organic carbon (TOC) loading (3.40 kgTOC·m^−3^·day^−1^), TOC removal rate (~95%), and hydraulic retention time (19.4 hours) over eight years. The wastewater contains 2,752 mgTOC·L^−1^ composed of TA, 4.5 mM; BZ, 6.0 mM; isophthalic acid (IA), 2.1 mM; orthophthalic acid (OA), 1.0 mM; PT, 0.6 mM; trimellitic acid (TMA), 1.0 mM; methanol (MT), 24.3 mM; acetate (AC), 35.7 mM; and methyl acetate (MA), 1.2 mM. The 12L mesophilic (38°C) lab-scale reactor U1 treating synthetic PTA wastewater has been operated for 11 months with the same hydraulic retention time and TOC loading rate as the full-scale reactors. The U1 reactor achieved a TOC removal rate of ~94% from wastewater with 1,700 mgTOC·L^−1^ containing TA, 3.6 mM; BZ, 2.5 mM; IA, 0.6 mM; OA, 0.3 mM; PT, 4.4 mM; TMA, 0.3 mM; MT, 4.8 mM; AC, 23.0 mM; and MA, 1.3 mM. The effluents of two full-scale reactors and a lab-scale reactor contained 390 and 86 mgSS·L^−1^, respectively.

### Analytical methods

The following parameters were measured daily on influent and effluent of both full-scale reactors: TOC was analyzed using a TOC analyzer (TOC-L CPN Basic System, Shimadzu, Japan). TSS was measured following the procedure of APHA (1998) [19]. For U1: Methanol and methyl acetate were detected using gas chromatography with FID on an HP5890 with a RTX-1 nonpolar column. Acetate is detected on a hp 5890 Series II with a DB-Wax polar column; and TA, BZ, IA, OA, PT, and TMA were detected using an Agilent 1200 HPLC System with multiple wavelength detector, or equivalent; the HPLC column was an Agilent SB-C18, 4.6 mm i.d. x 50 mm, 1.8 μm particle diameter (p/n 822975–902). For reactors E and F: TA, BZ, IA, OA, PT, and TMA were aromatic compounds, fatty acids and methyl compounds were detected using a high-performance liquid chromatography (Agilent ZORBAX Eclipse XDB-C18, Rapid Resolution HT 4.6 mm i.d. x50 mm, 1.8 μm particle diameter, operated at 600 Bar).

### Sample collection and DNA extraction

This study collected 300 granules with diameters from small (GSA: 1–2 mm), medium (GSB: 2–3 mm), to large (GSC: 3–4 mm) individually at two different depths (height: 1 m and 6 m) from full-scale reactors E and F and from lab-scale reactor U1. These granules were stored at -80°C until extraction of DNA. DNA was extracted from individual 300 granules by using FastDNA Spin Kit for Soil (MP Biomedicals, Carlsbad, CA, USA), according to the manufacturer’s protocol. Sludge samples used in this study were taken from UASB at IL, USA by permission from the BP America.

### PCR amplification and 16S rRNA gene sequencing

16S rRNA gene amplification was performed with the universal forward primer (Univ515F: 5’-GTGCCAGCMGCCGCGGTAA-3’) and the universal reverse primer (Univ909R: 5’-CCCCGYCAATTCMTTTRAGT-3’) [20, 21]. The PCR reaction (25 μL) containing 30 ng template DNA, 0.5 μM of forward and reverse primers, and 12.5 μL of Taq DNA polymerase 2.0 mix (Bulls eye, St Louis, MO, USA) was carried out using a thermal cycler (T100^™^, BIO-RAD, USA) with the following conditions: initial denaturation at 95°C for 3min, denaturation at 95°C for 45 s, annealing at 55°C for 60 s, elongation at 72°C for 90 s, and a final extension at 72°C for 10 min. The PCR cycle numbers were 25 cycles. PCR products were purified using Wizard SV Gel and PCR Clean-Up System (Promega, Fitchburg, WI, USA) according to manufacturer’s protocol. The 16S rRNA gene sequencing was conducted using the MiSeq Reagent kit v3 and MiSeq system (illumina, San Diego, CA, USA) at the Roy J. Carver Biotechnology Center at the University of Illinois at Urbana-Champaign.

### Data analysis

Raw 16S rRNA gene sequences were analyzed using QIIME ver. 1.8.0 [22]. The Phred quality score under 30 was trimmed using a fastx_trimmer tool (http://hannonlab.cshl.edu/fastx_toolkit/) before assembling with the paired-end assembler [23]. OTUs were selected with ≥97% sequence identity cut-off using the UCLUST [24]. Representative sequences of picked OTUs were aligned by PyNAST [25]. Chimeric sequences were identified from the alignments by ChimeraSlayer [26]. Taxonomy was assigned using blast retained on the Greengenes database ver. 13_8 [27]. Taxonomic placements of predominant OTUs were confirmed using the web-based Blast search (http://blast.ncbi.nlm.nih.gov/Blast.cgi) and the ARB program package based on Greengenes 16S rRNA gene database [27, 28]. The phylogenetic tree of 16S rRNA gene sequences was constructed based on neighbor-joining and parsimony methods in ARB using Greengenes 16S rRNA gene database [28]. The topology of constructed tree was confirmed by 1,000 bootstrap replicates [29]. We defined the OTU frequency on basis of its abundance and occurrence in individual granules. If one OTU occurred in ≥70% of sampled granules with >0.5% abundance, regardless of granule sources, we chose it as a predominant OTU. Finally, we decided core organisms with > 1.0% average abundance rate from the predominant OTUs.

### Statistical analysis

Alpha diversity indices (observed OTUs, Chao1, singles, doubles, phylogenetic diversity, and Good’s coverage) and the weighted UniFrac distances were calculated by QIIME. Chao1, singles, doubles, and phylogenetic diversity were calculated at a sampling depth of 18,000 reads. The weighted UniFrac distances were used for PCoA and jackknife-resampling methods (even sampling at 18,000 reads). Significant differences of alpha diversity indices were calculated using Welch’s t-test. The statistical analysis of metagenomic profiles software package was used to determine statistical differences of OTUs abundance [30]. To confirm the possible OTU interactions, we calculated Spearman’s rank correlation coefficients based on predominant and high frequent OTUs in reactor E [31]. For reduction of complexity, we chose the thresholds with Spearman’s correlation *rs* >0.4 and statistically significant p-value <0.001, respectively. The nodes and edges were used by representative taxa of each OTU and Spearman’s correlation, respectively. The node size shifts correspond to average OTU abundance. The OTU networks were visualized by using Cytoscape [32].

### Deposition of DNA sequence data

The raw 16S rRNA gene sequences deposited into the DDBJ Sequence Read Archive database (DRA005103). The 16S rRNA gene sequences of representative OTUs deposited into the DDBJ/EMBL/GenBank databases (LC177557–LC177598).

## Results and Discussion

To examine the heterogeneity of microbial community structure within individual granules, 300 individual granules with diameters greater than 1mm were collected individually from one lab-scale (U1) and two identical full-scale (E and F) UASB reactors and categorized into groups defined via granule size (GSA: 1-2mm, GSB: 2-3mm, and GSC: 3-4mm). The microbial community was analyzed by extracting community genomic DNA from each granule, amplifying 16S rRNA gene, and finally sequencing the amplicons using MiSeq Illumina sequencing technology, generating a total of 32 million 16S rRNA sequences with an assembled length of 374bp. For each granule, we obtained 18,900–158,000 sequences representing 420–1,452 operational taxonomic units (OTUs) based on a 97% 16S rRNA gene sequence similarity cut-off (Table A in S1 File), which adequately represent the majority of the microbial communities as indicated by calculated Chao1 indices (625–2,933 OTUs per sample) and Good’s coverage (≥0.99 under all conditions) (Table A in S1 File).

### Comparison of full- and lab- scale granules

**Fig 1:**
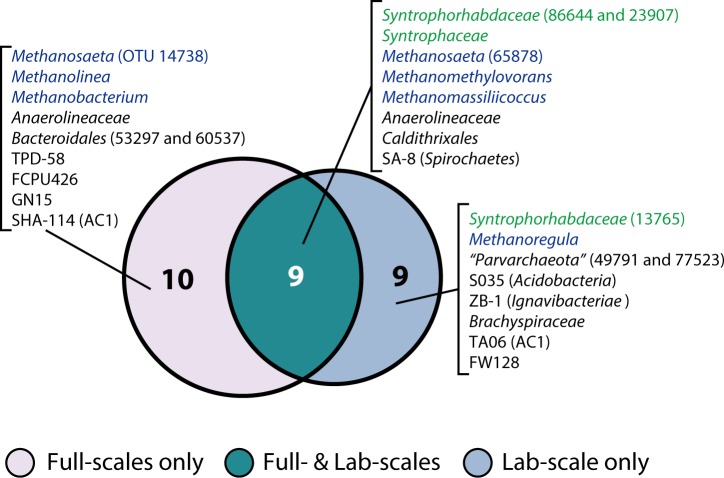
Venn diagram of the shared microorganisms in PTA-watewater treatment UASB granules. Syntrophs and methanogens are highlighted by green and blue, respectively.

As a first step in defining the granule microbial communities, we define core microbial populations in the studied full- and lab-scale reactors (>0.5% abundance in ≥70% granules in each reactor and > 1.0% average abundance rate; Figure B in S1 File). Treatment of the major PTA wastewater components (*e*.*g*., TA, BZ, and *para*-toluic acid–PT) require interaction between three organisms (*i*.*e*., syntrophs, acetate-degrading methanogens, and H_2_-oxidizing methanogens) [33], so we centralize our analysis around these niches. As expected, we detect consistent syntrophic aromatic compound degraders (*i*.*e*., *Syntrophorhabdus* OTUs 86644 and 23907 and *Syntrophus* OTU 57595) along with a partnering acetate-degrading methanogen (*Methanosaeta* OTU 14738) across all reactors (Fig 1, and Figure B and Table C in S1 File). On the other hand, the H_2_-oxidizing methanogens differ between the full-scale (*Methanolinea* OTU 43922 and *Methanobacterium* OTU 35610) and lab-scale (*Methanoregula* OTU 43922) reactors. Although fine physiological differences between phylogenetically distinct methanogens remain to be addressed, the ecology revolving around syntrophy and thermodynamics (*e*.*g*., H_2_ transfer) may be more different between full- and lab-scale reactors than previously expected. In addition to these methanogens, we also observe consistent methylotrophic methanogens (*Methanomethylovorans* OTU 70689 and *Methanomassiliicoccus* OTU 73432) responsible for degrading wastewater methanol to CH_4_.

Besides syntrophs and methanogens, we consistently detect populations related to uncultivated organisms with unknown function: *Anaerolineaceae*, *Bacteroidales*, *Spirochaetes*, TPD-58, FCPU426, GN04, AC1, FW128, and “*Candidatus* Parvarchaeota” (Fig 1 and Figure B in S1 File). Interestingly, “*Ca*. Parvarchaeota” members are specifically abundant in the lab-scale reactor. Although the ecological roles and metabolic capacity of these organisms remain unclear, their ubiquity across various methanogenic bioreactors suggests that further characterization of such organisms is necessary [4, 6, 7, 34–36]. In a later section, we perform network analyses to define the relationships of these organisms with syntrophs and methanogens.

### Co-existing granule types in full-scale UASB

**Fig 2:**
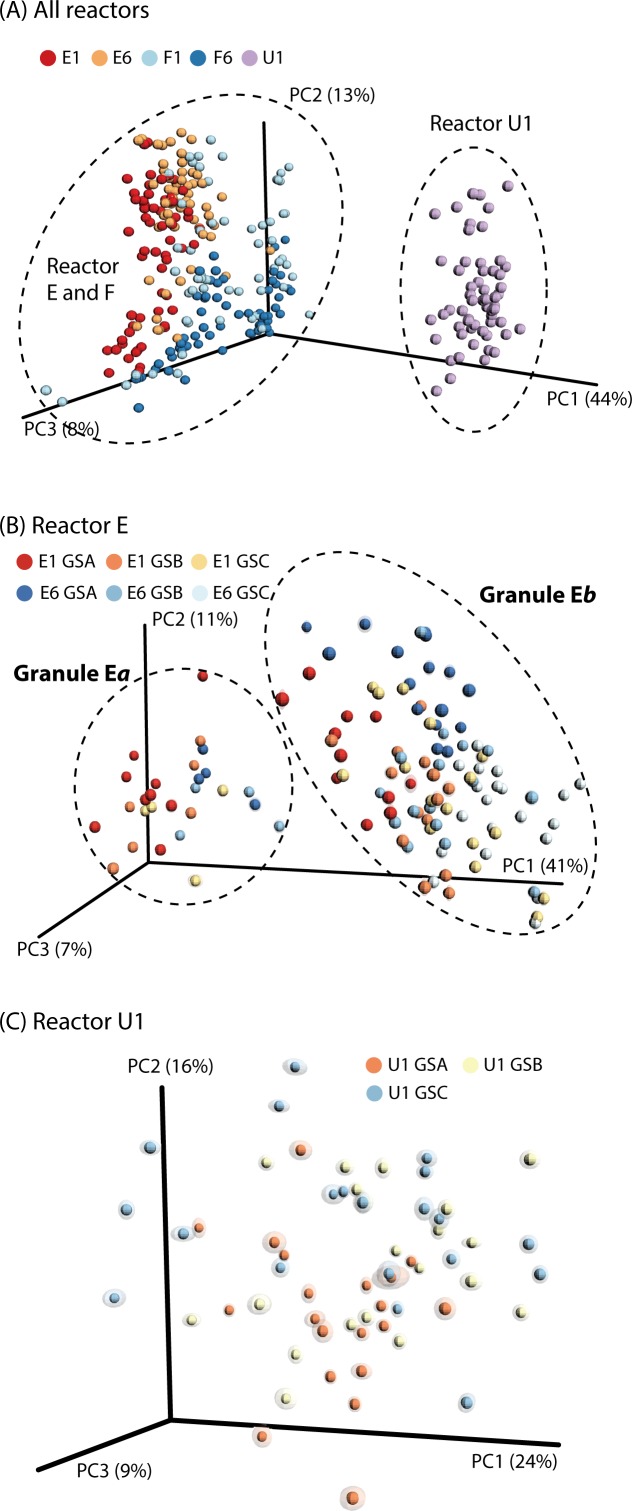
Jackknife-supported Principal coordinate analysis plots with weighted UniFrac in (A) All reactors, (B) reactor E, and (C) reactor U1. GSA, GSB, and GSC indicate small (1–2 mm), medium (2–3 mm), large (3–4 mm), respectively. For these analyses, 16S rRNA sequence reads were normalized to 18,000 reads per sample. “Cluster” of each granule type is supported by Jackknife-supported weighted UniFrac tree (S1 File).

**Fig 3:**
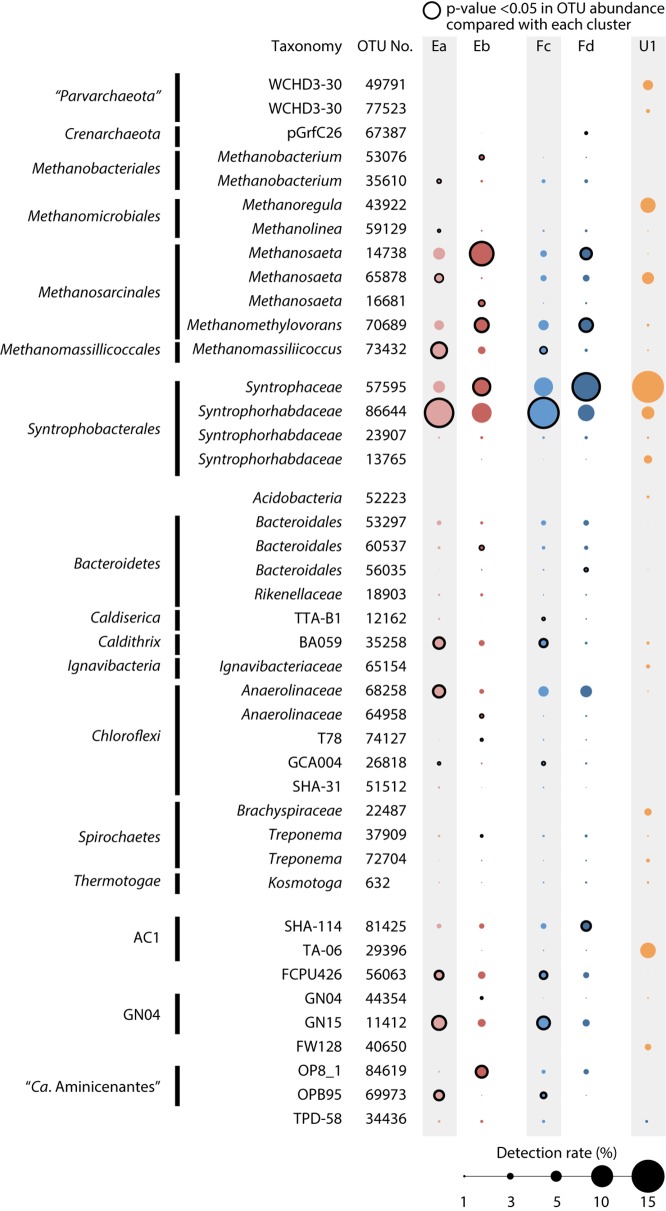
Abundance of predominant OTUs in reactors E, F and U1 using bubble plots. *Circle sizes* correspond to abundance rate, as shown at the bottom of the figure. *Circle lines* indicate the statistical differences of OTU abundance between different granule types based on Welch’s t-test (p<0.05).

Based on our hypothesis, the ecological roles and importance of core organisms (defined above) and satellite organisms may vary between individual granules, whereby creating a much more complex ecosystem that previously anticipated. Comparison of individual granules through principal coordinate analyses (PCoA) revealed high diversity among granules in full-scale (E and F; Fig 2A) and high homogeneity in lab-scale (U1; Fig 2C). Interestingly, differences among granules could neither be attributed to granule size (R^2^ = 0.85–0.99, Table B in S1 File) nor reactor depth (E1 *v*. E6: R^2^ = 0.94 and F1 *v*. F6: R^2^ = 0.94), suggesting that granule growth and vertical heterogeneity are not significant factors influencing the observed granule diversity. Much to our surprise, Jackknife-supported PCoA reveals that each full-scale reactor has two distinct and co-existing granule types regardless of different granule sizes and sampling depths (E1*a v*. E6*a*: R^2^ = 0.97, E1*b v*. E6*b*: R^2^ = 0.97, F1*c v*. F6*c*: R^2^ = 0.92, and F1*d* v. F*6*d: R^2^ = 0.87) (Fig 2 and Figure A in S1 File), though less distinct and poorly supported for reactor F (Figures A and E in S1 File). Moreover, the two co-existing granule types have different microbial populations dominating each niche (Fig 3).

In the reactor E, although the two granule types (E*a* and E*b*) host similar members, the abundance of organisms representing key niches is significantly different. In group E*a* and E*b*, different syntrophs predominate (*Syntrophorhabdus* 86644 in E*a* and *Syntrophus* 57595 respectively) (Fig 3 and Figure C in S1 File), suggesting that the ecology revolving around syntrophic aromatic compound degradation is different between the granule types. Corresponding to this, the dominant hydrogenotrophic (*Methanobacterium* 35610 *v*. 53076) and aceticlastic methanogens (*Methanosaeta* OTUs 65878 *v*. 16681) are different between the granule types. Interestingly, we observe a methanogen that simultaneously degrades H_2_ and methyl compounds (*Methanomassiliicoccus* OTU 73432) predominates in E*a*, while a methanogen specialized in methyl degradation (*Methanomethylovorans* 70689) is much more abundant in E*b*. The reactor F granule types F*c* and F*d* show a similar pattern for OTUs related to syntrophs and methylotrophic methanogens. Although reactor E and F are replicate reactors, granules E*a* and E*b* show clear differences in their microbial composition (R^2^ = 0.674 in OTU abundance scatter diagrams) while granules F*c* and F*d* are significantly more similar (R^2^ = 0.799) (Table D in S1 File). When comparing the granule types between reactors, the less abundant types in each reactor (E*a* and F*c)* have similar microbial community composition (*i*.*e*., R^2^ = 0.906); however, the more abundant granules (E*b* and F*d*) do not coincide (R^2^ = 0.768) (Table D in S1 File). Surprisingly, these results implicate that granules diverge into different types and develop differently in reactors with similar operation and identical feed. Although *Pelotomaculum* are associated with aromatic compound degradation and frequently found in PTA-wastewater treatment bioreactors [13], they were only found at average abundance rates below the threshold (0.85% (E*a*), 0.62% (E*b*), 0.67% (F*c*), 0.79% (F*d*), and 0.92% (U1)) set for the definition of core populations (average > 1.0%) (Figure C in S1 File). Besides, *Syntrophorhabdus* OTU86644 predominated the full- and lab-scale reactors (>10% and >5% respectively) as the major TA degrader, suggesting that *Pelotomaculum* may play less significant role in aromatic compound degradation in these reactors.

**Fig 4:**
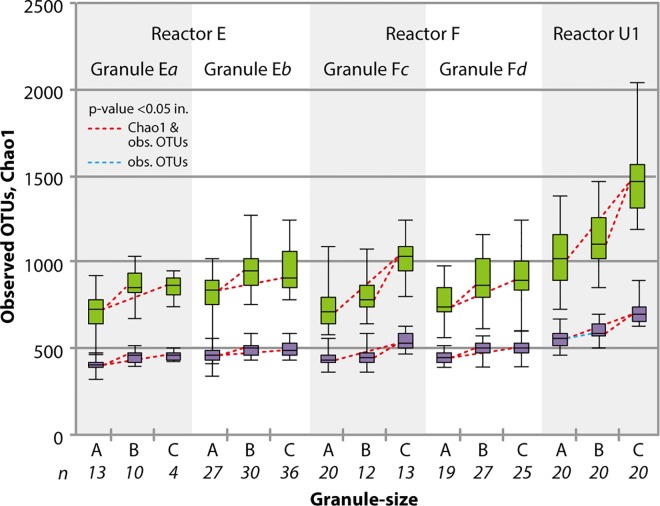
Boxplots of observed OTUs (lower box plots) and Chao1 (upper box plots). The dash lines indicate the statistical differences based on unpaired Welch’s t-test. The gray and black lines are p-value <0.05 in Chao1 and observed OTUs and p-value <0.05 in observed OTUs.

Within each granule type, we observe positive correlation between granule size and biodiversity based on alpha diversity indices (Fig 4 and Table A in S1 File). While the abundance and diversity of key degraders (*i*.*e*., syntrophs and methanogens) remains relatively consistent across different granule sizes (Figure C and Table B in S1 File), the diversity of low-abundance microbial populations with unknown ecological functions increases with increasing granule size. We suspect such organisms contribute to degrading and recycling detrital biomass [13] accumulated in the larger granules. Indeed, as granule size increases, poor substrate penetration into the inner layers leads to cell death, cell debris degradation, and development of a hollow core or inactive layer [4, 37–39]. Thus, while the observations are quite logical, the influence of such organisms on the ecology of the granules remains unclear.

### Distinct network of key microbial populations

**Fig 5:**
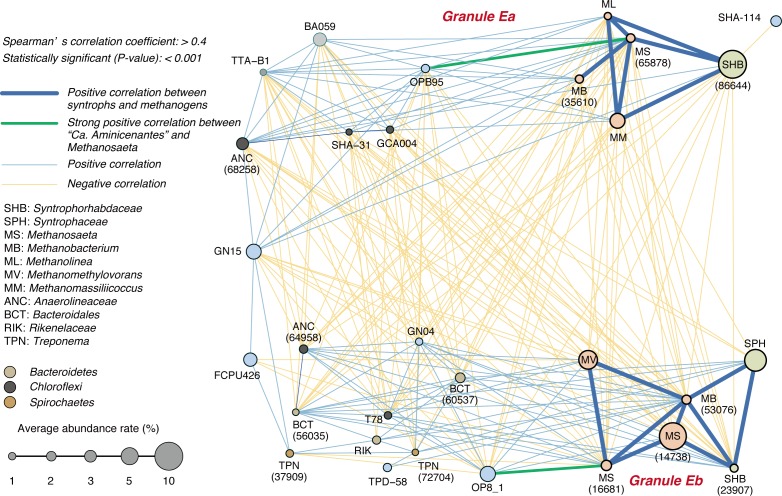
Network of predominant microorganisms in reactor E based on Spearman’s correlation analysis(Spearman’s *rs* > 0.4 and p-value < 0.001). Highlighted blue and green lines indicate the positive correlations between methanogens and syntrophs and strong positive correlations between “*Candidatus* Aminicenantes” and *Methanosaeta* (*rs* > 0.7), respectively. Light blue and light orange lines indicate the positive and negative correlations, respectively. *Circle sizes* correspond to average abundance rate, as shown at the left bottom of the figure. Orange circle color shows the methanogens nodes including *Methanosaeta* (MS), *Methanomethylovorans* (MV), *Methanobacterium* (MB), *Methanolinea* (ML), and *Methanomassiliicoccus* (MM). Light green circle color shows the syntrophs nodes including *Syntrophaceae* (SPH) and *Syntrophorhabdacea* (SHB). *Bacteroidales* (BCT), *Rikenelaceae* (RIK), and *Treponema* (TPN) were indicated with light brown, dark gray, and brown circle colors, respectively.

In order to identify specific microbial interactions between syntrophs, methanogens, and uncultivated organisms in the distinct granule types found in reactor E, microbial network analysis was performed based on Spearman’s rank correlation test (Fig 5). The analysis shows two sub-networks of microbial correlation reflecting granule types E*a* and E*b* (Figures C and E in S1 File). We observe positive correlations between syntrophs and methanogens highly abundant in granule E*a*: *Syntrophorhabdus* 86644 and two hydrogenotrophic methanogens (*Methanomassiliicoccus* 73432 and *Methanolinea* 59129) (Fig 5). Likewise, syntrophs and methanogens associated with granule type E*b* share positive correlation: *Syntrophus* 57595 and *Syntrophorhabdus* 23907 with *Methanobacterium* 53076. Perhaps in granule type E*a*, syntrophs prioritize direct syntrophic association with multiple H_2_-utilizing methanogens [33]; on the other hand, the unexpected correlation between two syntrophic aromatic compound degraders in E*b* suggests that E*b*-associated syntrophs may form inter-syntroph interactions (“secondary syntrophy”) (*e*.*g*., *Syntrophorhabdus* degrades TA to BZ and/or butyrate and *Syntrophus* further degrades these byproducts) [14, 40, 41]. Differences in syntrophic interactions suggest that the thermodynamic ecology may differ between granule types E*a* and E*b*. Perhaps changes in the thermodynamic environment between granules led to shifts in the microbial community, granule structure, and ultimately microbial interactions.

Besides syntrophs, different “*Ca*. Aminicenantes” populations (OTUs 84619 and 69973) predominate in each granule type (Fig 3) and each population correlates with different aceticlastic methanogens (Fig 5; OTUs 69973 and 65878, *rs* = 0.72; OTUs 84619 and 16681, *rs* = 0.82). Based on their suspected capacity to degrade amino acids [42, 43], “*Ca*. Aminicenantes” populations may ferment detrital amino acids, provide acetate to specific *Methanosaeta*, and potentially contribute to controlling which aceticlastic methanogen populations establish in different granule types. Thus, uncultivated organisms may play significant roles in the development and fate of granular sludge in UASB reactors.

## Conclusion

By performing in-depth single-granule-level microbial community analyses, we show for the first time that multiple consortiums of microorganisms can coexist in different granules within the same full-scale bioreactor. Based on these results, we also provide evidence that microbial interactions between syntrophs, methanogens, and uncultivated organisms may differ between granules in the same reactor. More specifically, the thermodynamic environment may slightly differ between different types granules. In addition, syntrophs and uncultivated organisms may contribute to controlling which hydrogenotrophic and aceticlastic methanogens establish and predominate in the granules. Further 16S rRNA gene analysis of other full-scale reactors employing the same approach will provide hitherto overlooked insight into granule heterogeneity and development in UASB reactors. Moreover, metagenomic investigation of uncultivated groups like “*Ca*. Aminicenantes” will advance our understanding of microbial interactions driving methanogenic treatment of various wastewater types. In total, through application of a novel single-granule level approach, we could address unseen microbial relationships and granule characteristics in the UASB reactors.

## Supporting Information

S1 File **Figure A in S1 File. Jackknife-supported weighted UniFrac tree for 16S rRNA gene-based granule community in (A) reactor E, (B) reactor F, and (C) reactor U1. GSA, GSB, and GSC indicate the granule diameter as 1–2 mm, 2–3 mm, and 3–4 mm, respectively. For this analysis, 16S rRNA sequence reads were normalized to 18,000 reads per sample. The solid circle, open circle, and open squera indicate the Jackknife-supported probabilities at >75%, >50%, and >25%, respectively.** This is the Figure A legend in S1 File. **Figure B in S1 File. Phylogenetic tree representing predominant OTUs in PTA-wastewater treatment UASB reactor using the neighbor-joining and parsimony methods based on 16S rRNA gene sequences. The solid circle, open circle, and open squera indicate the bootstrap-supported probabilities at >90%, >75%, and >50%, respectively. Circle colors of OTU frequency indicate the OTUs existence patterns such as core OTU in PTA wastewater treatment (red), core in full-scale (green), core in lab-scale (yellow), and others (blue).** This is the Figure B legend in S1 File. **Figure C in S1 File. Abundance of predominant OTUs in (A) reactors E, (B) reactor F, and (C) reactor U1 with different sized granules using bubble plots. GSA, GSB, and GSC show the granule diameter as 1–2 mm, 2–3 mm, and 3–4 mm, respectively. *Circle sizes* correspond to abundance rate, as shown at the bottom of the figure. Asterisk indicates *Pelotomaculum* OTU, which is not core organism in this study.** This is the Figure C legend in S1 File. **Figure D in S1 File. Extended error bar plot with significant different OTUs abundances (p<0.05) in reactor E and F.** This is the Figure D legend in S1 File. **Figure E in S1 File. Jackknife-supported Principal coordinate analysis (PCA) plots with weighted UniFrac in reactor F. GSA, GSB, and GSC indicate small (1–2 mm), medium (2–3 mm), large (3–4 mm), respectively. For these analyses, 16S rRNA sequence reads were normalized to 18,000 reads per sample. “Cluster” of each granule type is supported by Jackknife-supported weighted UniFrac tree (Figure A). No overlap between granule F*d* and F*c* are observed in the PCA plots.** This is the Figure E legend in S1 File. **Table A in S1 File. Alpha diversity indices in PTA wastewater treatment UASB granules.** This is the Table A legend in S1 File. **Table B in S1 File. The Coefficient of determination based on OTU scatter diagram of different sized granule in each granule type.** This is the Table B legend in S1 File. **Table C in S1 File. Taxonomic assignment of representative OTUs of this study.** This is the Table C legend in S1 File. **Table D in S1 File. The coefficient of determination based on OTUs scatter diagram of each granule type.** This is the Table D legend in S1 File.
(PDF)
