# Supplementary material for: A Single-Granule-Level Approach Reveals Ecological Heterogeneity in an Upflow Anaerobic Sludge Blanket Reactor
Source: PLoS One. 2016 Dec 9;11(12):e0167788. doi: 10.1371/journal.pone.0167788 (PMC5147981; doi:10.1371/journal.pone.0167788)
Supplement: S1 File — Figure A in S1 File. Jackknife-supported weighted UniFrac tree for 16S rRNA gene-based granule community in (A) reactor E, (B) reactor F, and (C) reactor U1. GSA, GSB, and GSC indicate the granule diameter as 1–2 mm, 2–3 mm, and 3–4 mm, respectively. For this analysis, 16S rRNA sequence reads were normalized to 18,000 reads per sample. The solid circle, open circle, and open squera indicate the Jackknife-supported probabilities at >75%, >50%, and >25%, respectively. This is the Figure A legend in S1 File. Figure B in S1 File. Phylogenetic tree representing predominant OTUs in PTA-wastewater treatment UASB reactor using the neighbor-joining and parsimony methods based on 16S rRNA gene sequences. The solid circle, open circle, and open squera indicate the bootstrap-supported probabilities at >90%, >75%, and >50%, respectively. Circle colors of OTU frequency indicate the OTUs existence patterns such as core OTU in PTA wastewater treatment (red), core in full-scale (green), core in lab-scale (yellow), and others (blue). This is the Figure B legend in S1 File. Figure C in S1 File. Abundance of predominant OTUs in (A) reactors E, (B) reactor F, and (C) reactor U1 with different sized granules using bubble plots. GSA, GSB, and GSC show the granule diameter as 1–2 mm, 2–3 mm, and 3–4 mm, respectively. Circle sizes correspond to abundance rate, as shown at the bottom of the figure. Asterisk indicates Pelotomaculum OTU, which is not core organism in this study. This is the Figure C legend in S1 File. Figure D in S1 File. Extended error bar plot with significant different OTUs abundances (p<0.05) in reactor E and F. This is the Figure D legend in S1 File. Figure E in S1 File. Jackknife-supported Principal coordinate analysis (PCA) plots with weighted UniFrac in reactor F. GSA, GSB, and GSC indicate small (1–2 mm), medium (2–3 mm), large (3–4 mm), respectively. For these analyses, 16S rRNA sequence reads were normalized to 18,000 reads per sample. “Cluster” of each granule type [file pone.0167788.s001.pdf]

A single-granule-level approach reveals ecological heterogeneity in an upflow anaerobic sludge blanket reactor

– Supporting Information–

Kyohei Kuroda[1,2¶], Masaru K. Nobu[1¶], Ran Mei[1], Takashi Narihiro[1,3],
Benjamin T.W. Bocher[4], Takashi Yamaguchi[2], and Wen-Tso Liu[1*]

[1]Department of Civil and Environmental Engineering, University of Illinois at Urbana-Champaign, 205 North Mathews Ave, Urbana, Illinois 61801, United States of America
[2]Department of Environmental systems Engineering, Nagaoka University of Technology, 1603-1, Kami-tomioka, Nagaoka, Niigata 940-2188, Japan
[3]Bioproduction Research Institute, National Institute of Advanced Industrial Science and Technology (AIST), Central 6, Higashi 1-1-1, Tsukuba, Ibaraki 305-8566, Japan
[4]Petrochemicals Technology, BP America, 150 West Warrenville Road Building 300, Naperville, Illinois 60563, United States of America
[¶]These authors contributed equally to this work.

[*]Corresponding author:
Wen-Tso Liu
Department of Civil and Environmental Engineering
University of Illinois at Urbana-Champaign
205 North Mathews Ave, Urbana, Illinois 61801, United States of America
Tel: +1 217 333 8442; E-mail: wtliu@illinois.edu

(A)

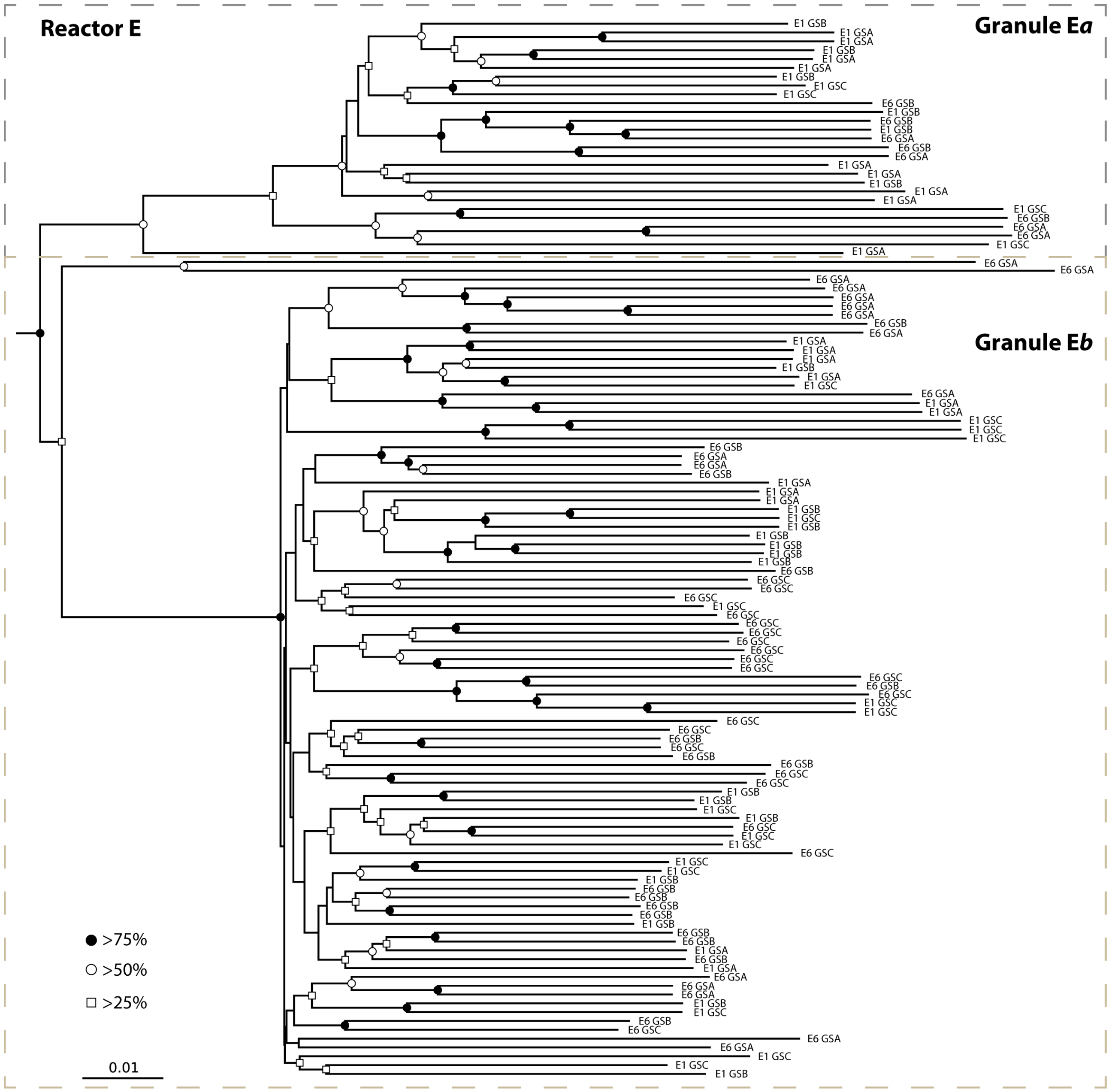
Reactor E
Granule Ea
E1 GSB
E1 GSA
E1 GSA
E1 GSB
E1 GSA
E1 GSA
E1 GSB
E1 GSC
E1 GSC
E6 GSB
E1 GSB
E6 GSB
E1 GSB
E6 GSA
E6 GSB
E6 GSA
E1 GSA
E1 GSA
E1 GSB
E1 GSA
E1 GSA
E1 GSC
E6 GSB
E6 GSA
E6 GSA
E1 GSC
E1 GSA
E6 GSA
E6 GSA
Granule Eb
E6 GSA
E6 GSA
E6 GSA
E6 GSA
E6 GSA
E6 GSB
E6 GSA
E1 GSA
E1 GSA
E1 GSA
E1 GSB
E1 GSA
E1 GSC
E6 GSA
E1 GSA
E1 GSA
E1 GSC
E1 GSC
E1 GSC
E6 GSB
E6 GSA
E6 GSA
E6 GSB
E1 GSA
E1 GSA
E1 GSA
E1 GSB
E1 GSC
E1 GSB
E1 GSB
E1 GSB
E1 GSB
E1 GSB
E6 GSB
E6 GSC
E6 GSC
E6 GSC
E1 GSC
E6 GSC
E6 GSC
E6 GSC
E6 GSC
E6 GSC
E6 GSC
E6 GSC
E6 GSC
E6 GSB
E6 GSC
E1 GSC
E1 GSC
E6 GSC
E6 GSC
E6 GSB
E6 GSC
E6 GSB
E6 GSB
E6 GSC
E6 GSC
E1 GSB
E1 GSB
E1 GSC
E1 GSB
E6 GSC
E1 GSC
E1 GSC
E6 GSC
E1 GSC
E1 GSC
E1 GSB
E6 GSB
E6 GSB
E6 GSB
E6 GSB
E1 GSB
E6 GSB
E6 GSB
E1 GSA
E6 GSB
E1 GSA
E6 GSA
E6 GSA
E6 GSA
E1 GSB
E1 GSC
E6 GSB
E6 GSC
E6 GSA
E6 GSA
E1 GSC
E1 GSC
E1 GSB
>75%
>50%
>25%
0.01

(Continued)

(B)

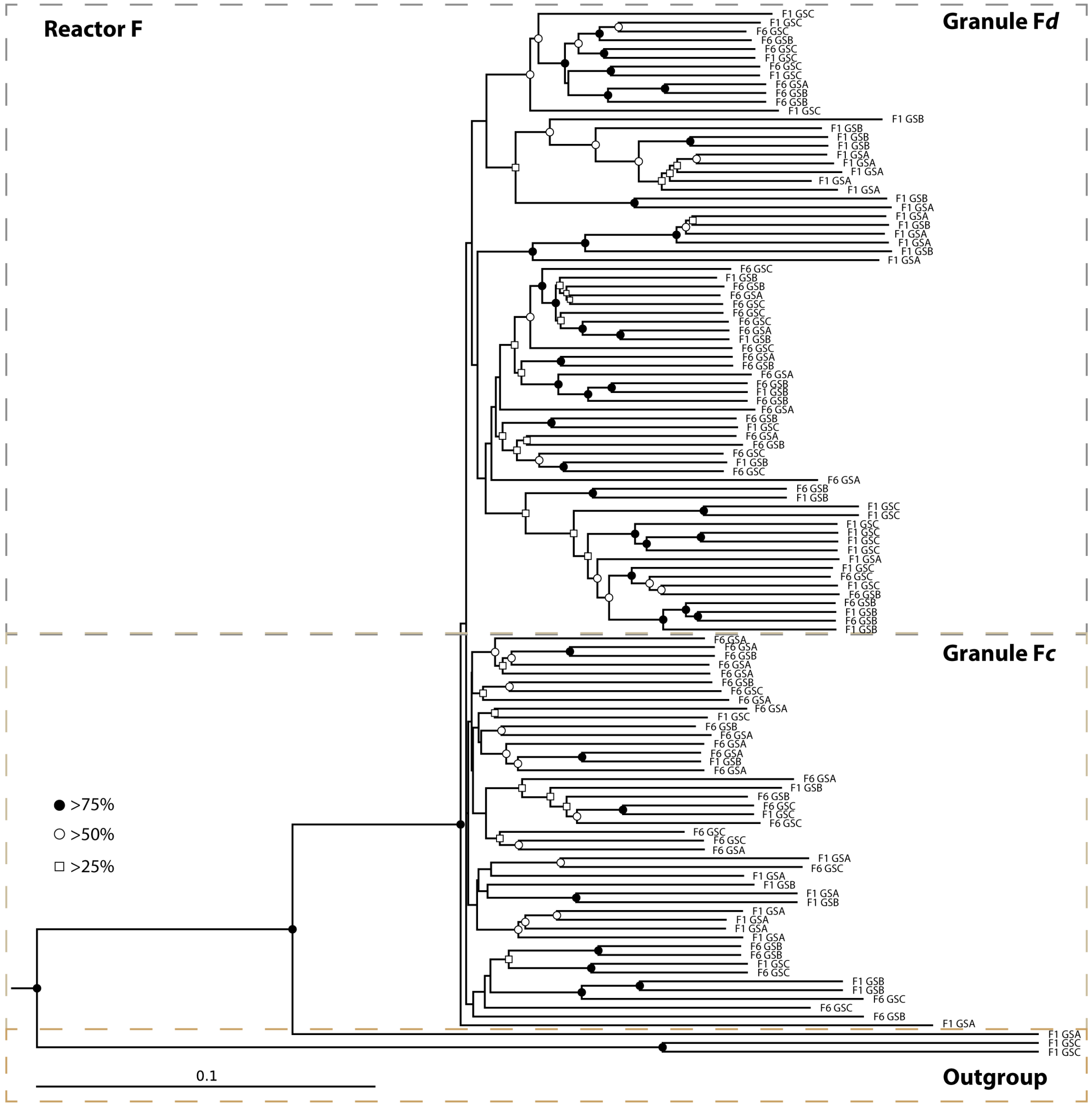

(Continued)

(C)

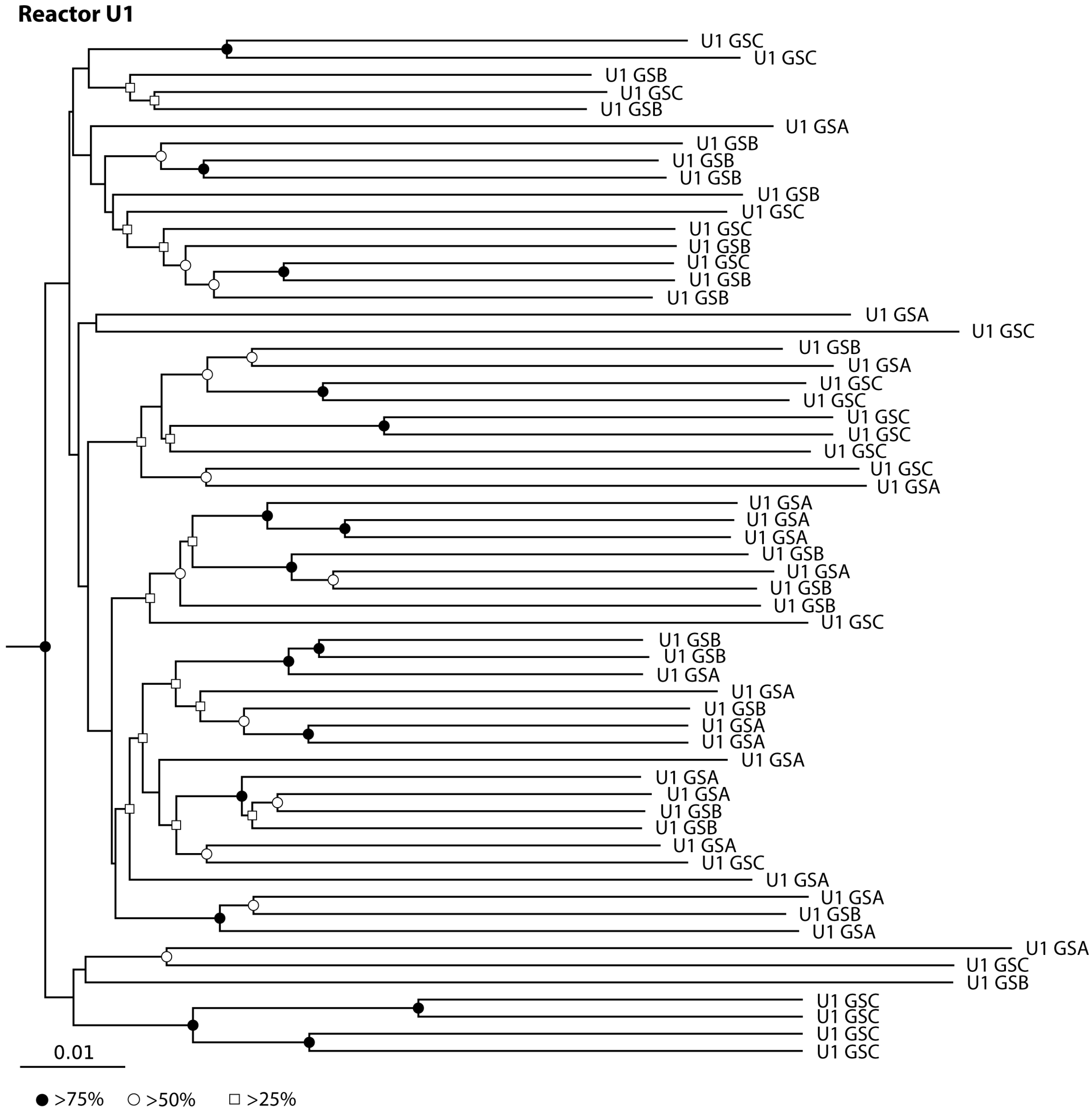

Figure A. Jackknife-supported weighted UniFrac tree for 16S rRNA gene-based granule community in (A) reactor E, (B) reactor F, and (C) reactor U1. GSA, GSB, and GSC indicate the granule diameter as 1–2 mm, 2–3 mm, and 3–4 mm, respectively. For this analysis, 16S rRNA sequence reads were normalized to 18,000 reads per sample. The solid circle, open circle, and open squera indicate the Jackknife-supported probabilities at >75%, >50%, and >25%, respectively.

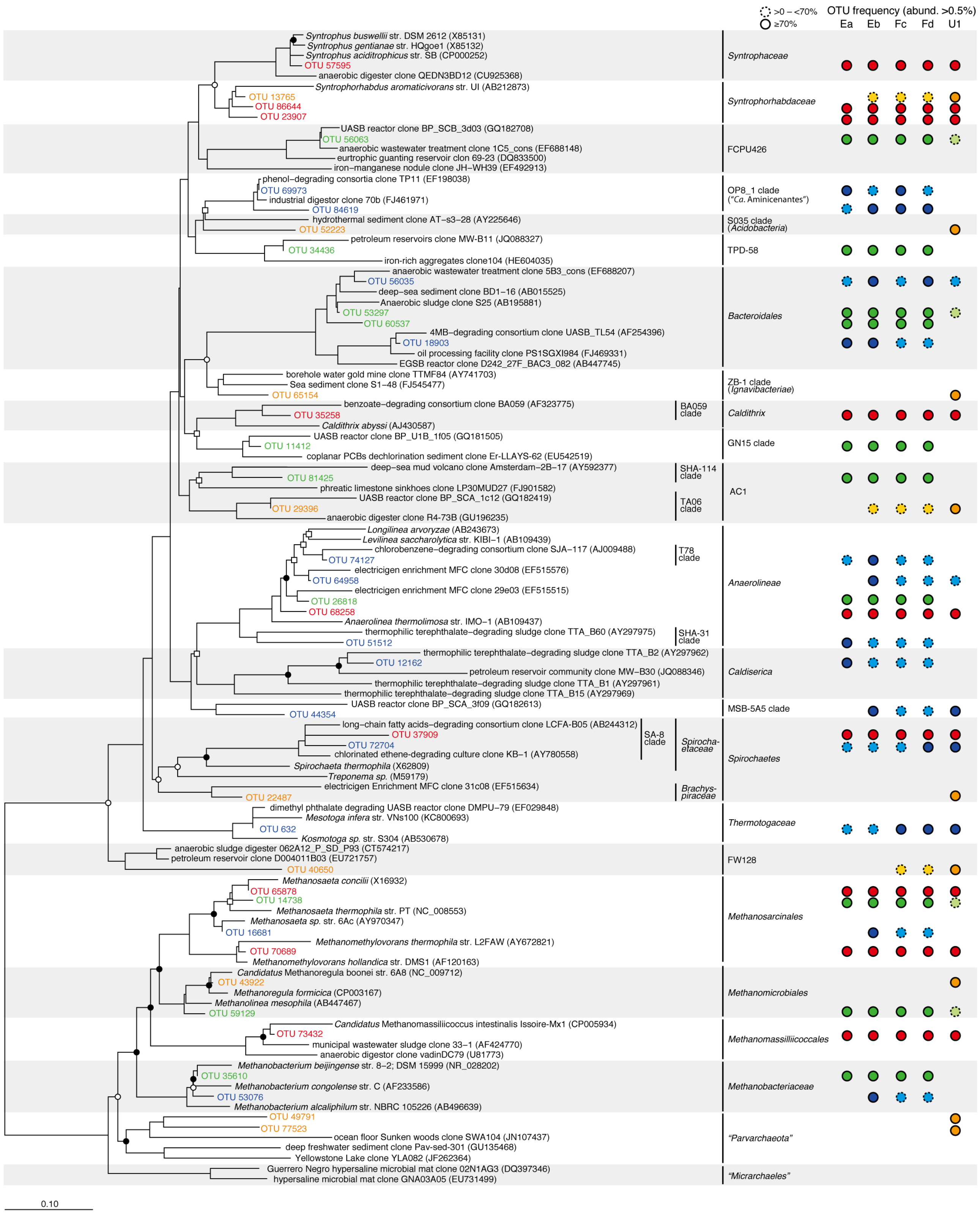

Figure B. Phylogenetic tree representing predominant OTUs in PTA-wastewater treatment UASB reactor using the neighbor-joining and parsimony methods based on 16S rRNA gene sequences. The solid circle, open circle, and open squera indicate the bootstrap-supported probabilities at >90%, >75%, and >50%, respectively. Circle colors of OTU frequency indicate the OTUs existence patterns such as core OTU in PTA wastewater treatment (red), core in full-scale (green), core in lab-scale (yellow), and others (blue).

(A)

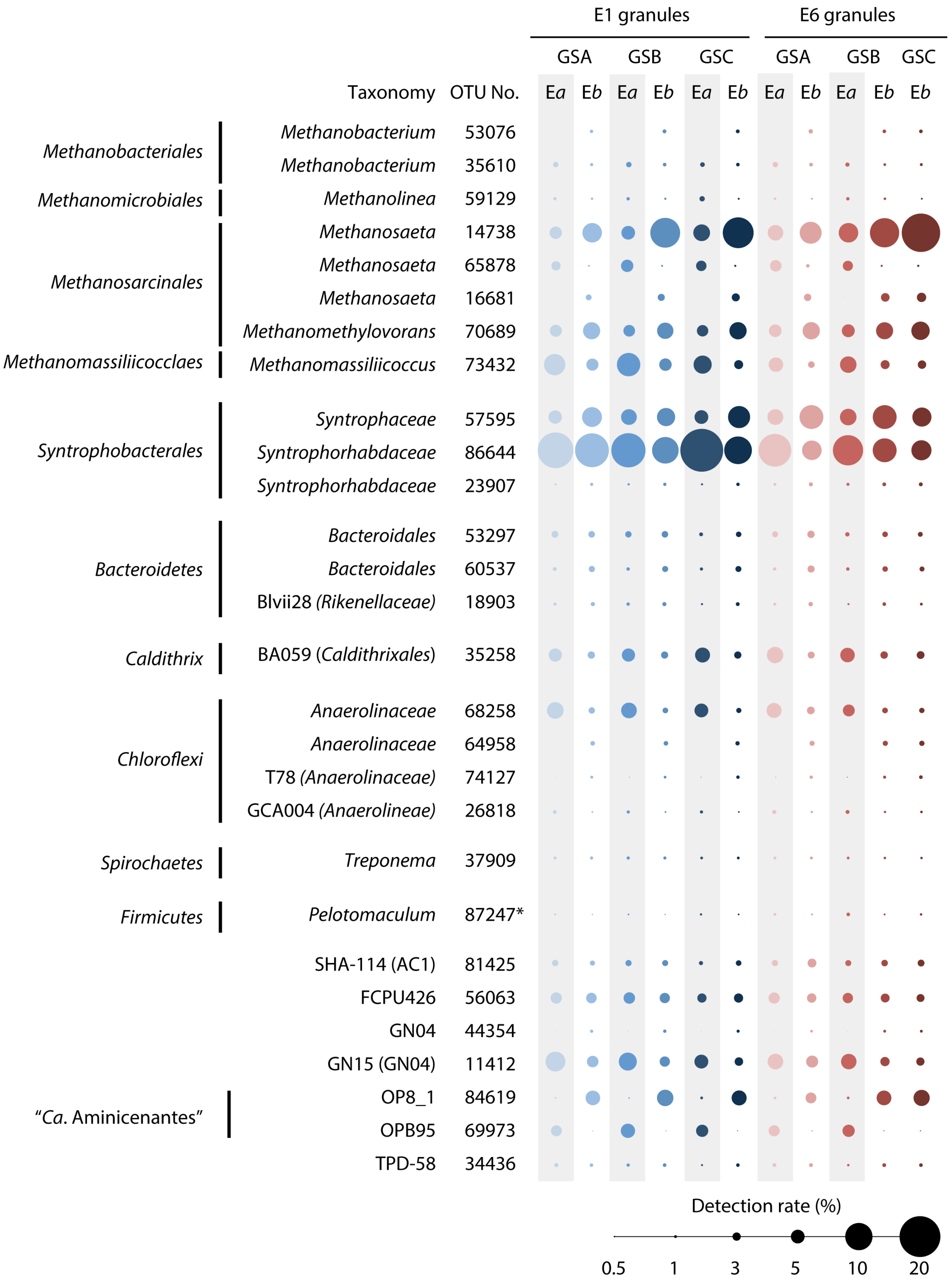
E1 granules
E6 granules
GSA
GSB
GSC
GSA
GSB
GSC
Taxonomy
OTU No.
Ea Eb Ea Eb Ea Eb Ea Eb Ea Eb Eb
Methanobacteriales
Methanobacterium 53076
Methanobacterium 35610
Methanomicrobiales
Methanolinea 59129
Methanosarcinales
Methanosaeta 14738
Methanosaeta 65878
Methanosaeta 16681
Methanomethylovorans 70689
Methanomassiliicocclaes
Methanomassiliicoccus 73432
Syntrophobacterales
Syntrophaceae 57595
Syntrophorhabdaceae 86644
Syntrophorhabdaceae 23907
Bacteroidetes
Bacteroidales 53297
Bacteroidales 60537
Blvii28 (Rikenellaceae) 18903
Caldithrix
BA059 (Caldithrixales) 35258
Chloroflexi
Anaerolinaceae 68258
Anaerolinaceae 64958
T78 (Anaerolinaceae) 74127
GCA004 (Anaerolineae) 26818
Spirochaetes
Treponema 37909
Firmicutes
Pelotomaculum 87247*
SHA-114 (AC1) 81425
FCPU426 56063
GN04 44354
GN15 (GN04) 11412
"Ca. Aminicenantes"
OP8_1 84619
OPB95 69973
TPD-58 34436
Detection rate (%)
0.5
1
3
5
10
20

(Continued)

(B)

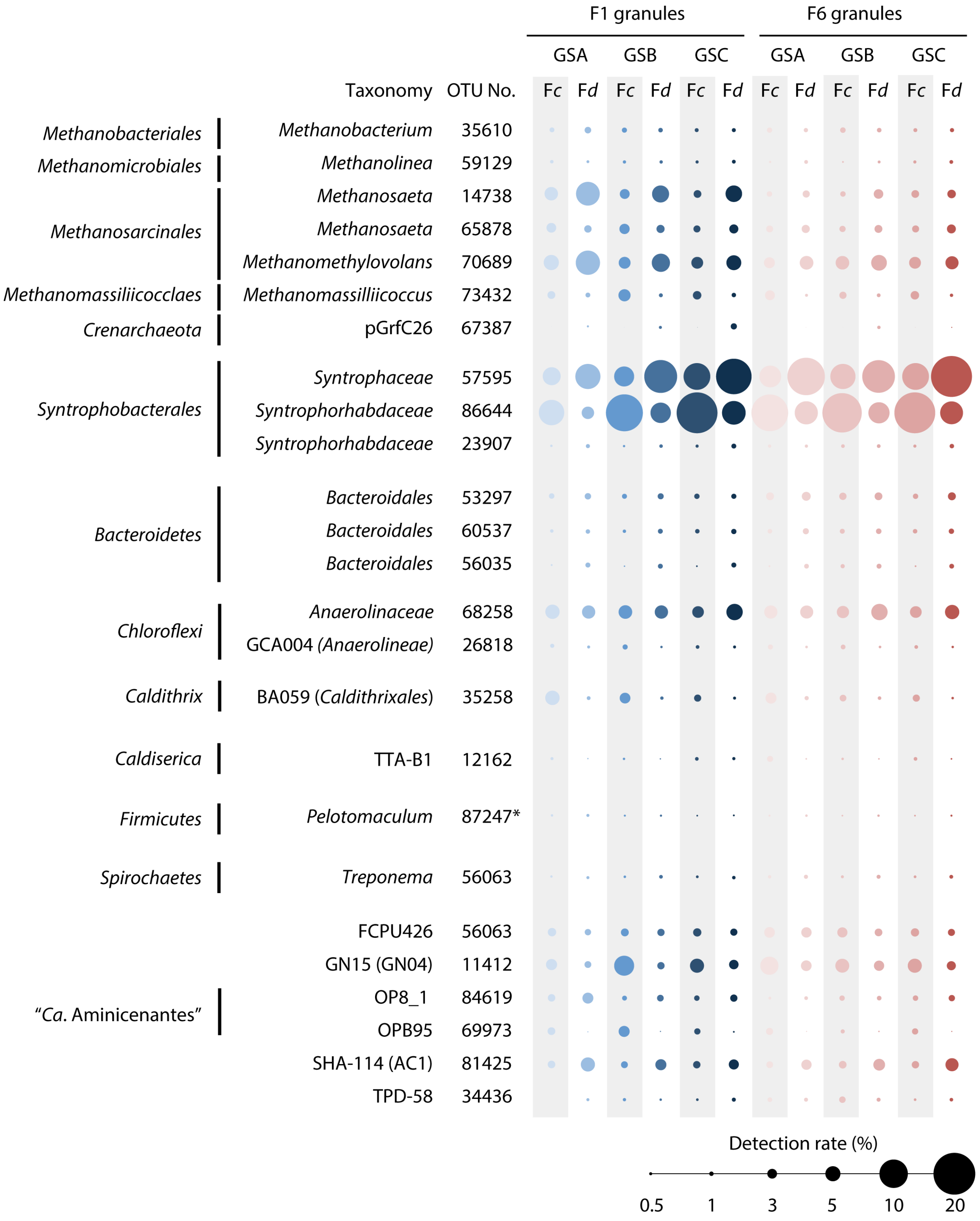
F1 granules
F6 granules
GSA
GSB
GSC
GSA
GSB
GSC
Taxonomy
OTU No.
Fc
Fd
Fc
Fd
Fc
Fd
Fc
Fd
Fc
Fd
Fc
Fd
Methanobacteriales
Methanobacterium
35610
Methanomicrobiales
Methanolinea
59129
Methanosarcinales
Methanosaeta
14738
Methanosaeta
65878
Methanomethylovolans
70689
Methanomassiliicocclaes
Methanomassilliicoccus
73432
Crenarchaeota
pGrfC26
67387
Syntrophobacterales
Syntrophaceae
57595
Syntrophorhabdaceae
86644
Syntrophorhabdaceae
23907
Bacteroidetes
Bacteroidales
53297
Bacteroidales
60537
Bacteroidales
56035
Chloroflexi
Anaerolinaceae
68258
GCA004 (Anaerolineae)
26818
Caldithrix
BA059 (Caldithrixales)
35258
Caldiserica
TTA-B1
12162
Firmicutes
Pelotomaculum
87247*
Spirochaetes
Treponema
56063
"Ca. Aminicenantes"
FCPU426
56063
GN15 (GN04)
11412
OP8_1
84619
OPB95
69973
SHA-114 (AC1)
81425
TPD-58
34436
Detection rate (%)
0.5
1
3
5
10
20

(Continued)

(C)

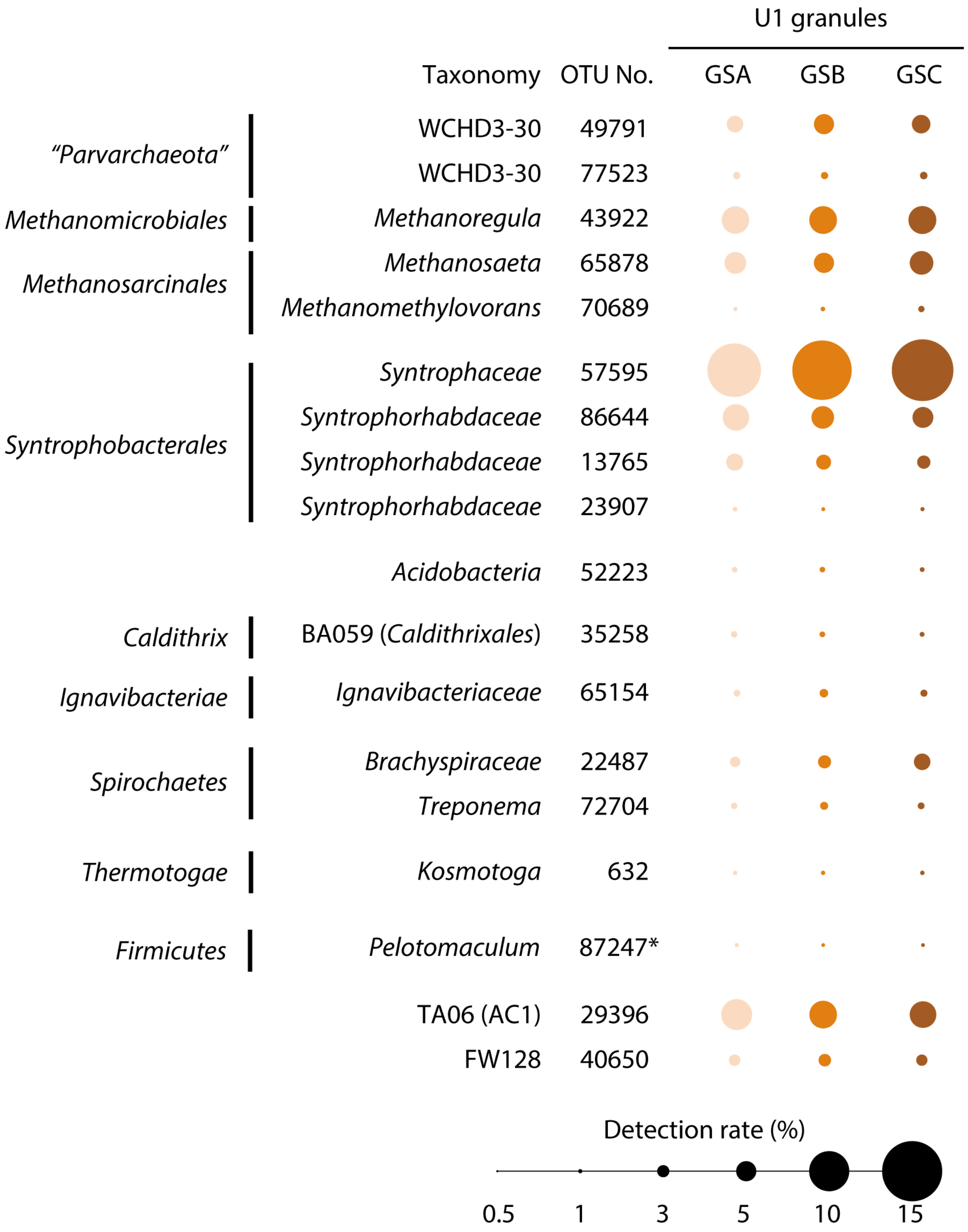

Figure C. Abundance of predominant OTUs in (A) reactors E, (B) reactor F, and (C) reactor U1 with different sized granules using bubble plots. GSA, GSB, and GSC show the granule diameter as 1–2 mm, 2–3 mm, and 3–4 mm, respectively. *Circle sizes* correspond to abundance rate, as shown at the bottom of the figure. Asterisk indicates *Pelotomaculum* OTU, which is not core organism in this study.

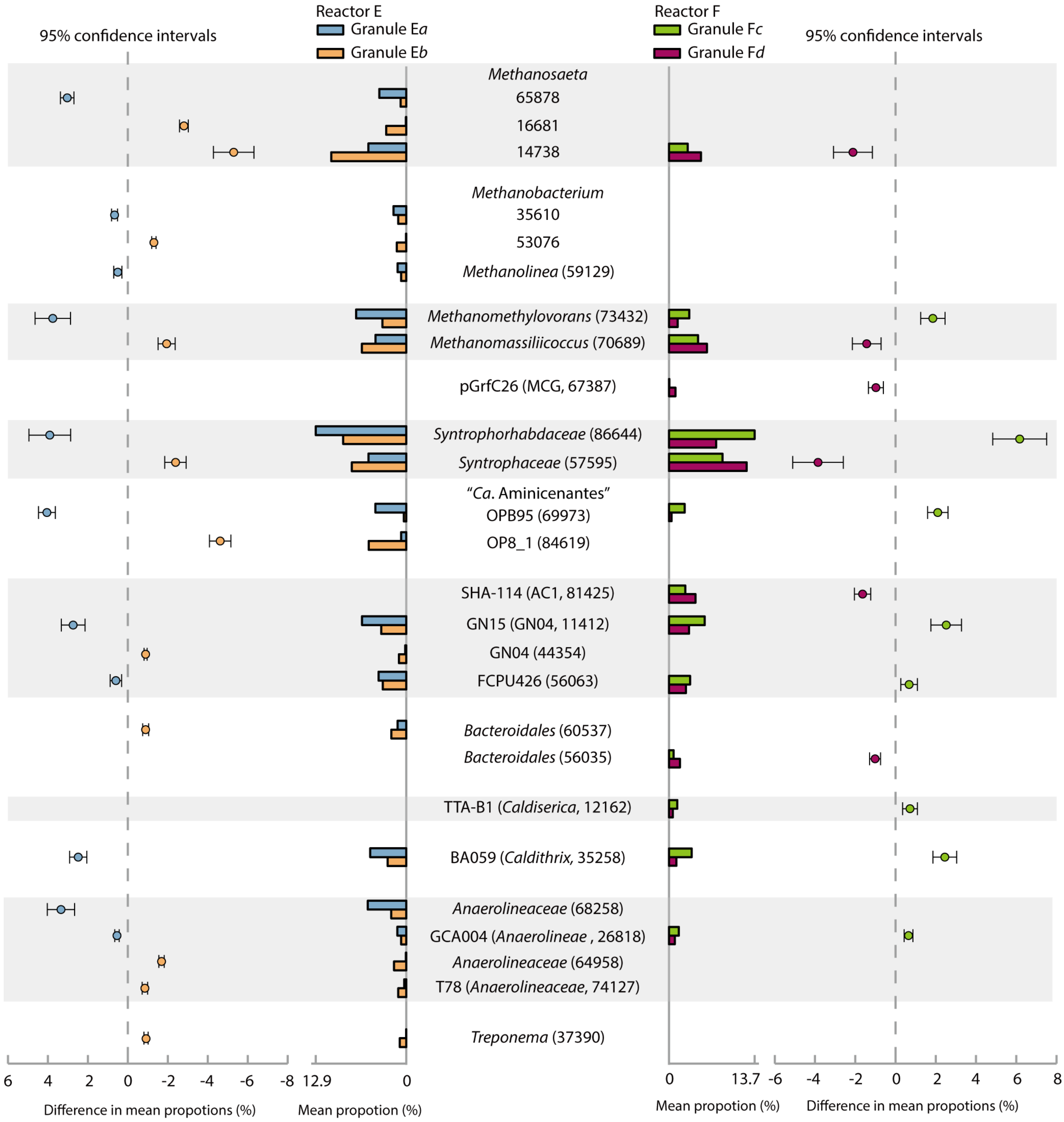

Figure D. Extended error bar plot with significant different OTUs abundances ($p<0.05$) in reactor E and F.

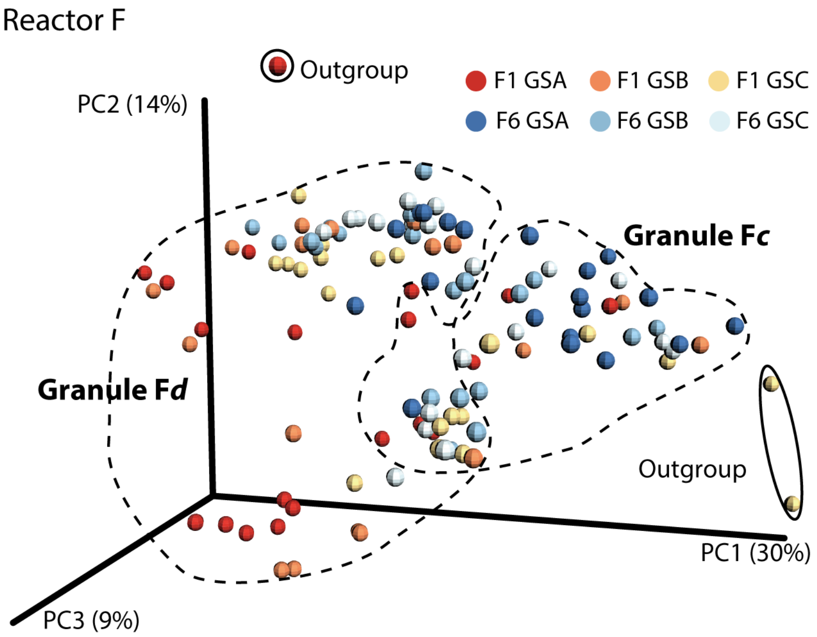

Figure E. Jackknife-supported Principal coordinate analysis plots with weighted UniFrac in reactor F. GSA, GSB, and GSC indicate small (1–2 mm), medium (2–3 mm), large (3–4 mm), respectively. For these analyses, 16S rRNA sequence reads were normalized to 18,000 reads per sample. “Cluster” of each granule type is supported by Jackknife-supported weighted UniFrac tree (Figure A). No overlap between granule F*d* and F*c* are observed in the PCA plots.

Table A. Alpha diversity indices in PTA wastewater treatment UASB granules.

Reactor Name	granule	Granular size	No. of granules	Diversity Indices[¶]						
				No. of Seq	No. of OTUs	Chao1[¶¶]	Singles[¶¶]	Doubles[¶¶]	PD[§¶¶]	Coverage
E	E*a*	GSA[§§]	13	89020	400 ± 38	709 ± 117	172 ± 32	48 ± 6	75 ± 4	1.00
(Full-scale)		GSB*	10	87337	452 ± 39	865 ± 113	214 ± 29	56 ± 7	80 ± 5	0.99
		GSC**	4	81910	460 ± 35	855 ± 90	215 ± 20	58 ± 3	81 ± 4	0.99
	E*b*	GSA	27	90118	457 ± 48	821 ± 131	199 ± 36	55 ± 7	80 ± 5	1.00
		GSB	30	86879	495 ± 41	956 ± 132	233 ± 32	59 ± 6	82 ± 4	0.99
		GSC	36	80146	499 ± 46	954 ± 137	235 ± 33	61 ± 6	83 ± 5	0.99
F	F*c*	GSA	20	72992	440 ± 49	744 ± 130	180 ± 34	54 ± 7	82 ± 5	0.99
(Full-scale)		GSB	12	53555	453 ± 58	820 ± 129	202 ± 37	55 ± 10	80 ± 6	0.99
		GSC	13	35635	541 ± 56	1016 ± 120	256 ± 32	69 ± 9	88 ± 6	0.99
	F*d*	GSA	19	78661	451 ± 38	770 ± 118	183 ± 32	53 ± 6	82 ± 4	0.99
		GSB	27	55654	497 ± 41	895 ± 144	217 ± 38	60 ± 7	86 ± 4	0.99
		GSC	25	36616	510 ± 48	922 ± 151	225 ± 38	62 ± 7	87 ± 4	0.99
U1	–	GSA	20	44600	559 ± 53	1027 ± 181	248 ± 44	67 ± 7	94 ± 6	0.99
(Lab-scale)		GSB	20	42544	600 ± 56	1137 ± 177	282 ± 43	75 ± 7	97 ± 5	0.99
		GSC	20	42819	709 ± 71	1481 ± 224	371 ± 56	89 ± 10	105 ± 6	0.99

[¶]Calculations based on the operational taxonomic units (OTUs) determined at an evolutionary distance of 0.03

[¶¶]Calculation at a sampling depth of 18,000 reads

[§]Phylogenetic diversity

[§§]Range of Granular sludge diameter is 1.0–2.0 mm

*Range of Granular sludge diameter is 2.0–3.0 mm

**Range of Granular sludge diameter is 3.0–4.0 mm

Table B. The Coefficient of determination based on OTU scatter diagram of different sized granule in each granule type.

E1*a*	Granule	GSA	GSB	GSC		E6*a*	Granule	GSA	GSB	GSC
	GSA						GSA			
	GSB	0.99					GSB	0.98		
	GSC	0.95	0.95				GSC	–	–	
E1*b*	Granule	GSA	GSB	GSC		E6*b*	Granule	GSA	GSB	GSC
	GSA						GSA			
	GSB	0.95					GSB	0.96		
	GSC	0.94	0.99				GSC	0.89	0.96	
F1*c*	Granule	GSA	GSB	GSC		F6*c*	Granule	GSA	GSB	GSC
	GSA						GSA			
	GSB	0.91					GSB	0.98		
	GSC	0.88	0.98				GSC	0.97	0.98	
F1*d*	Granule	GSA	GSB	GSC		F6*c*	Granule	GSA	GSB	GSC
	GSA						GSA			
	GSB	0.94					GSB	0.98		
	GSC	0.85	0.94				GSC	0.95	0.91	

Table C. Taxonomic assignment of representative OTUs of this study.

OTU ID	*Greengenes ver. 13_8*		*Related species or clones in NCBI database*	
	Taxon		*identities (%)*	*Taxonomy (Accession No.)*
49791	*"Parvarchaeota"*	WCHD3-30	319/380(84%)	Uncultured euryarchaeote clone KuA23 (AB077233)
77523		WCHD3-30	245/253(97%)	Uncultured archaeon OTU_6052 (LN775620)
67387	*Crenarchaeota*	pGrfC26	321/380(84%)	*Candidatus* Nitrosocaldus yellowstonii strain HL72 (EU239960)
53076	*Euryarchaeota*	*Methanobacterium*	380/380(100%)	*Methanobacterium subterraneum* strain 9-7 (DQ649330)
35610		*Methanobacterium*	380/380(100%)	*Methanobacterium beijingense* strain 4-1 (AY552778)
43922		*Methanoregula*	376/380(99%)	*Methanoregula boonei* strain 6A8 (NR_074180)
59129		*Methanolinea*	376/380(99%)	*Methanolinea tarda* strain NOBI-1 (NR_028163)
14738		*Methanosaeta*	363/380(96%)	*Methanosaeta thermophila* strain PT (NR_074214)
65878		*Methanosaeta*	380/380(100%)	*Methanosaeta concilii* strain X16932 (KM408635)
16681		*Methanosaeta*	379/380(99%)	*Methanosaeta harundinacea* strain 6Ac (NR_102896)
70689		*Methanomethylovorans*	376/380(99%)	*Methanomethylovorans hollandica* strain DSM 15978 (NR_102454)
73432		*Methanomassiliicoccus*	377/379(99%)	*Candidatus* Methanomassiliicoccus intestinalis Issoire-Mx1 (CP005934)
57595	*Proteobacteria*	*Syntrophaceae*	373/376(99%)	*Syntrophus gentianae* strain HQGOe1 (JQ346737)
86644		*Syntrophorhabdaceae*	365/375(97%)	*Syntrophorhabdus aromaticivorans* UI (NR_041306)
23907		*Syntrophorhabdaceae*	359/375(96%)	*Syntrophorhabdus aromaticivorans* UI (NR_041306)
13765		*Syntrophorhabdaceae*	369/375(98%)	*Syntrophorhabdus aromaticivorans* UI (NR_041306)
52223	*Acidobacteria*	unassigned group	324/376(86%)	*Thermoanaerobaculum aquaticum* strain MP-01 (NR_109681)
53297	*Bacteroidetes*	*Bacteroidales*	335/372(90%)	*Prolixibacter bellariivorans* strain JCM 13498 (LC015091)
60537		*Bacteroidales*	328/372(88%)	*Ruminofilibacter xylanolyticum* strain S1 (DQ141183)
56035		*Bacteroidales*	341/374(91%)	*Prolixibacter bellariivorans* strain JCM 13498 (LC015091)
18903		*Rikenellaceae*	313/373(84%)	*Pontibacter korlensis* strain AG6 (KJ949605)
12162	*Caldiserica*	TTA-B1	311/376(83%)	*Caldisericum exile* strain AZM16c01 (NR_075015)
35258	*Caldithrix*	BA059	337/379(89%)	*Caldithrix palaeochoryensis* strain MC (NR_116885)
65154	*Ignavibacteriae*	*Ignavibacteriaceae*	341/374(91%)	*Melioribacter roseus* strain P3M-2 (NR_074796)
68258	*Chloroflexi*	*Anaerolineaceae*	352/375(94%)	*Leptolinea tardivitalis* strain YMTK-2 (NR_040971)
64958		*Anaerolineaceae*	350/375(93%)	*Longilinea arvoryzae* strain KOME-1 (NR_041355)
74127		T78	340/375(91%)	*Leptolinea tardivitalis* strain YMTK-2 (NR_040971)
26818		GCA004	348/375(93%)	*Leptolinea tardivitalis* strain YMTK-2 (NR_040971)
51512		SHA-31	314/378(83%)	*Anaerolinea thermolimosa* strain IMO-1 (NR_040970)
22487	*Spirochaetes*	*Brachyspiraceae*	313/374(84%)	*Exilispira thermophila* strain RASEN (NR_041644)
37909		*Treponema*	322/372(87%)	*Treponema zuelzerae* strain DSM (NR_104797)
72704		*Treponema*	316/372(85%)	*Treponema zuelzerae* strain DSM (NR_104797)
632	*Thremotogae*	*Kosmotoga*	375/375(100%)	*Mesotoga infera* strain VNs100 (NR_117646)
81425	AC1	SHA-114	361/368(98%)	Uncultured bacterium clone 6E1_cons (EF688249)
29396		TA-06	376/376(100%)	Uncultured bacterium clone BP_SCC_2c10 (GQ182963)
56063	FCPU426		376/376(100%)	Uncultured bacterium clone BP_SCC_2a10 (GQ182861)
44354	GN04	unassigned group	377/377(100%)	Uncultured bacterium clone BP_SCA_3d05 (GQ182494)
11412		GN15	376/376(100%)	Bacterium enrichment culture clone L11_2_64 (JX473550)
40650	OD1	unassigned group	370/376(98%)	Uncultured bacterium clone B16 (JX100399)
84619	"*Ca.* Aminicenantes"	OP8_1	374/374(100%)	Bacterium enrichment culture clone L55B-115 (JF947100)
69973		OPB95	374/374(100%)	Uncultured bacterium clone HMTAb196 (KM373094)
34436	TPD-58	unassigned group	376/376(100%)	Uncultured bacterium clone MW-B11 (JQ088327)

Table D. The Coefficient of determination based on OTU scatter diagram of each granule type.

Scatter R^2		E		F	
	Granlule	*a*	*b*	*c*	*d*
E	*a*		**0.674**	**0.906**	**0.64**
	b	**0.674**		**0.712**	**0.768**
F	*c*	**0.906**	**0.712**		**0.799**
	d	**0.64**	**0.768**	**0.799**	
